# Durable Marked Response of Unresectable Esophageal Squamous Cell Carcinoma After Radiotherapy Alone Followed by Combination Immunotherapy

**DOI:** 10.1155/crom/3970015

**Published:** 2026-02-27

**Authors:** Yasuhiro Watanabe, Shin′ichi Miyamoto, Yoichi Asano, Hiroaki Yaku, Kazuhito Ueki, Norio Araki, Taro Ueo

**Affiliations:** ^1^ Department of Gastroenterology, NHO Kyoto Medical Center, Kyoto, Japan; ^2^ Third Department of Internal Medicine, Kansai Medical University, Hirakata, Japan, kmu.ac.jp; ^3^ Clinical Research Institute, NHO Kyoto Medical Center, Kyoto, Japan; ^4^ Department of Radiation Oncology, NHO Kyoto Medical Center, Kyoto, Japan

**Keywords:** combination immunotherapy, esophageal cancer, ipilimumab, nivolumab, radiotherapy

## Abstract

We report the case of a 70‐year‐old man with unresectable esophageal squamous cell carcinoma who achieved durable marked response with combined immune checkpoint inhibitor (ICI) therapy following radiotherapy (RT) alone. The patient presented with dysphagia and stridor due to a bulky esophageal tumor and cervical lymph node metastasis compressing the trachea. He was diagnosed with cT3N1M0 (Stage IIIB; 8th edition of the TNM classification) or cT4(101R‐Trachea)N1M0 (Stage IVA; 12th edition of the Japanese classification). He underwent RT alone (total dose, 50 Gy), resulting in substantial tumor shrinkage and improvement in Eastern Cooperative Oncology Group performance status from 4 (due to ventilator dependence) to 1. Following confirmation of programmed death‐ligand 1 positivity (tumor proportion score ≧ 1%), treatment with nivolumab (anti**–**programmed cell death**-**1 antibody) and ipilimumab (anti–cytotoxic T‐lymphocyte antigen‐4 antibody) was initiated. The therapeutic effect was remarkable, and treatment continued for 14 courses until an immune‐related adverse event of secondary adrenal insufficiency interrupted therapy. Thereafter, there was no apparent recurrence on imaging for 31 months after treatment initiation. This case highlights the potential effect of RT followed by ICI combination therapy. Preclinical and clinical data suggest that prior RT may enhance systemic tumor immune responses. This therapeutic approach is currently being prospectively evaluated in the ongoing Phase II trial by the Japan Clinical Oncology Group (JCOG2311).

## 1. Introduction

Esophageal squamous cell carcinoma (ESCC) remains a disease with poor prognosis when diagnosed at an unresectable stage. Although immune checkpoint inhibitors (ICIs) have emerged as promising agents, early progression is common and compromises outcomes. A combination of nivolumab (anti‐programmed cell death [PD]‐1 antibody) plus ipilimumab (anti‐cytotoxic T‐lymphocyte antigen [CTLA]‐4 antibody) demonstrated superior overall survival (OS) compared with chemotherapy in the CheckMate 648 trial [1, 2]. Updated clinical guidelines also emphasize the role of ICIs in advanced gastroesophageal cancers [3]. Radiotherapy (RT) has been shown to enhance the efficacy of ICIs, as evidenced by the abscopal effect, that is, regression of tumors outside the irradiated field following ICI administration [4–7]. However, its synergistic effect has not been clearly demonstrated in clinical trials [8]. Moreover, the optimal RT dose, ICI regimen, and treatment sequence remain undefined. Here, we report a case of unresectable ESCC in which RT followed by combination immunotherapy with nivolumab and ipilimumab resulted in a durable and profound clinical response.

## 2. Case Presentation

**Figure 2:**
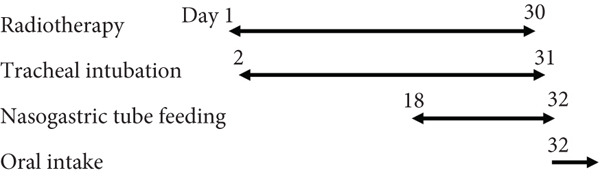
The treatment process during radiotherapy. Day 1 is defined as the initiation day of radiotherapy.

A 70‐year‐old man presented with progressive dysphagia and dyspnea. Endoscopic examination revealed a Type 1 tumor spanning 28–38 cm from the incisors (Figure 1a). Contrast‐enhanced computed tomography (CT) demonstrated circumferential wall thickening of the upper esophagus (Figure 1b, arrowhead) and cervical lymphadenopathy compressing the trachea (Figure 1c, arrowhead). Biopsy confirmed ESCC. He was diagnosed with cT3N1M0 (Stage IIIB; 8th edition of the TNM classification), or cT4(101R‐Trachea)N1M0 (Stage IVA; 12th edition of the Japanese classification). Because cervical lymph node metastasis was suspected to involve the main bronchus, the tumor was deemed unresectable. The patient underwent RT alone (2.5 Gy × 20 fractions, total 50 Gy), which was complicated by aspiration‐induced airway obstruction requiring intubation. RT was continued while the patient remained intubated and on nasogastric tube feeding. Details of treatment process during RT are shown in Figure 2. RT was effective, resulting in shrinkage of both the primary lesion (Figure 3a,b, arrowhead) and lymph node metastasis (Figure 3c, arrowhead). Compression of the trachea caused by lymph node metastasis resolved, allowing extubation at the completion of RT. Oral intake was resumed, and Eastern Cooperative Oncology Group performance status improved rapidly from 4 to 1. Given the tumor reduction achieved by RT, the risk of esophagotracheal fistula associated with cytotoxic chemotherapy, and the positive expression of programmed death‐ligand 1 (Clone 28‐8, tumor proportion score ≥ 1%), combination immunotherapy with nivolumab and ipilimumab was initiated 2 weeks after the completion of RT as a subsequent therapeutic strategy.

Figure 1Baseline findings. (a) Endoscopy showing a Type 1 esophageal tumor. (b) CT demonstrating circumferential wall thickening (arrowhead). (c) Cervical lymphadenopathy compressing the trachea (arrowhead).

**Figure:**
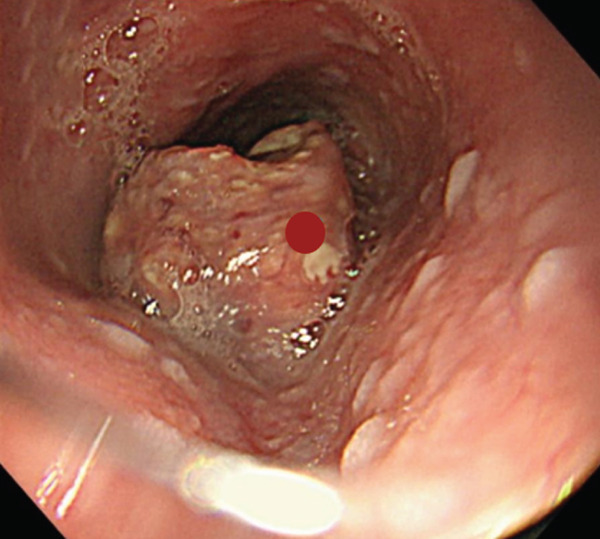
(a)

**Figure:**
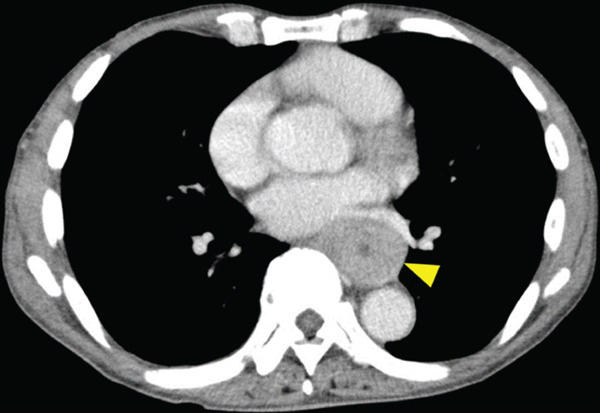
(b)

**Figure:**
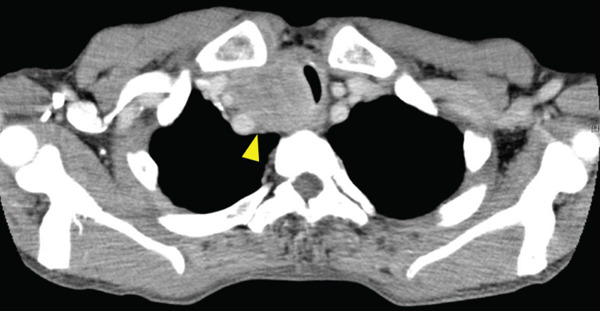
(c)

Figure 3Findings after radiotherapy alone (50 Gy). Shrinkage of the primary lesion on endoscopy (a) and CT (b, arrowhead). Reduction of cervical lymph node metastasis with resolution of tracheal compression (c, arrowhead).

**Figure:**
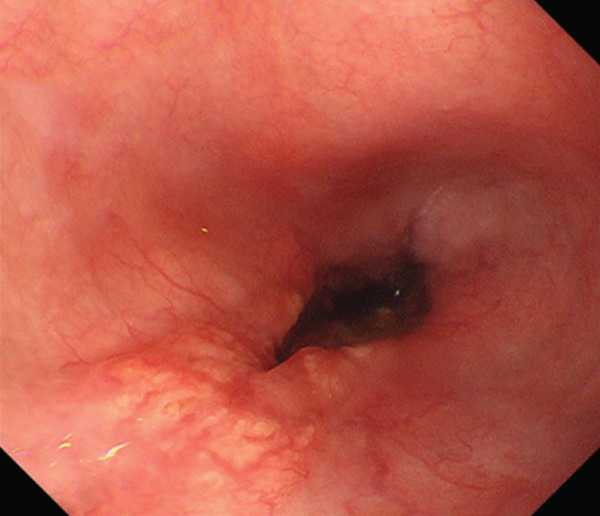
(a)

**Figure:**
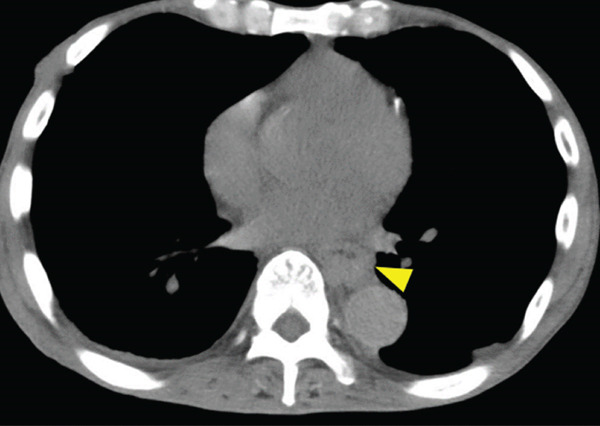
(b)

**Figure:**
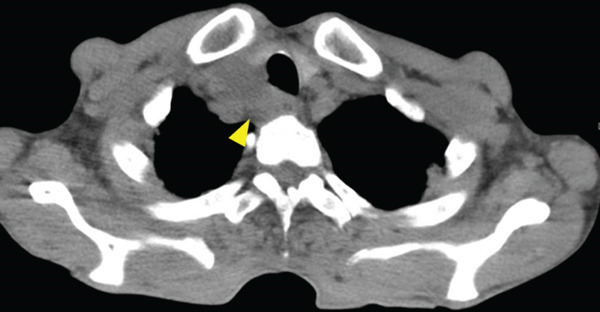
(c)

After one course of treatment, the primary tumor had completely disappeared endoscopically (single multipoint biopsies were negative) (Figure 4a). After three courses of treatment, the circumferential wall thickening of the upper esophagus (Figure 4b, arrowhead) and lymph node metastasis were markedly reduced on CT (Figure 4c, arrowhead). For convenience, the dosing schedule of nivolumab was modified to every 3 weeks from the fourth course onward and was continued accordingly.

Figure 4Findings after combination immunotherapy. Complete disappearance of the primary tumor on endoscopy after one course of treatment (a). On CT after three courses of treatment, marked reduction of the esophageal lesion (b, arrowhead) and lymph node metastasis (c, arrowhead).

**Figure:**
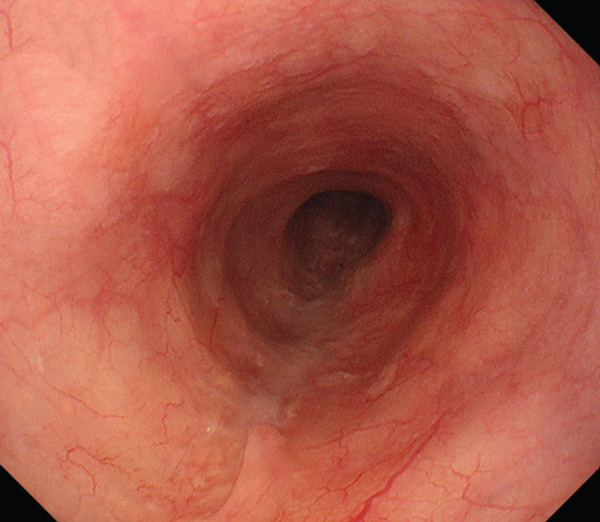
(a)

**Figure:**
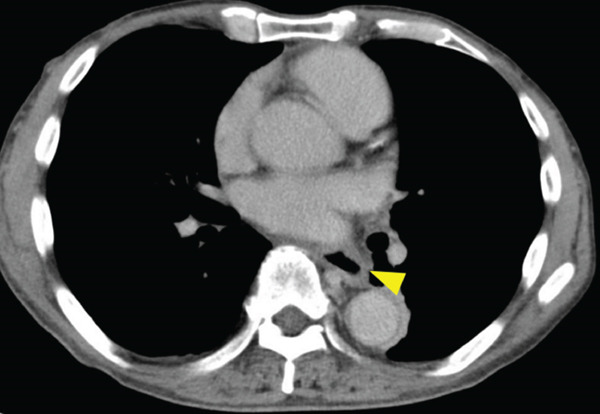
(b)

**Figure:**
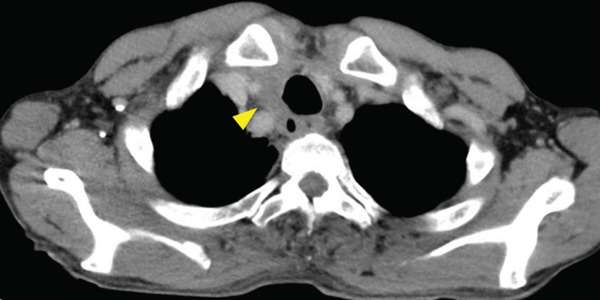
(c)

Thereafter, there was no apparent recurrence on imaging. Eighteen months after the initial treatment (after 14 courses of therapy were completed), the patient developed an immune‐related adverse event of secondary adrenal insufficiency, and steroid replacement therapy was started, after which treatment was discontinued. Figure 5a shows the endoscopic findings of the primary lesion at 3 months after completion of combination immunotherapy (23 months after treatment initiation), and CT findings of the primary lesion (Figure 5b, arrowhead) and lymph node metastasis (Figure 5c, arrowhead). Imaging follow‐up (EGD and CT) was performed after treatment discontinuation every 4 months with the final evaluation at 31 months. The patient is doing well without recurrence of disease.

Figure 5Findings at 23 months after treatment initiation. Endoscopy showing no recurrence (a). CT showing no apparent residual tumor in the esophagus (b, arrowhead) and lymph node (c, arrowhead).

**Figure:**
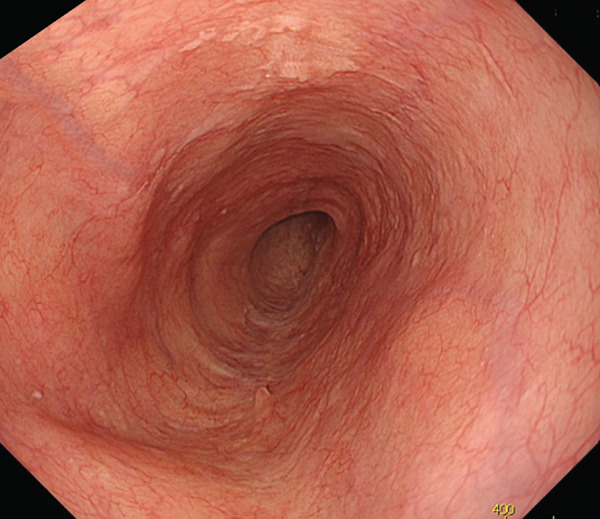
(a)

**Figure:**
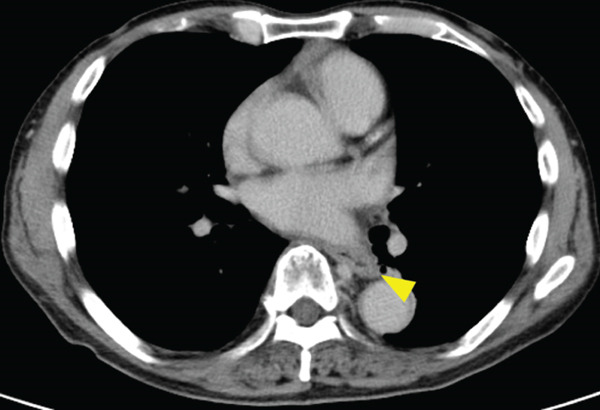
(b)

**Figure:**
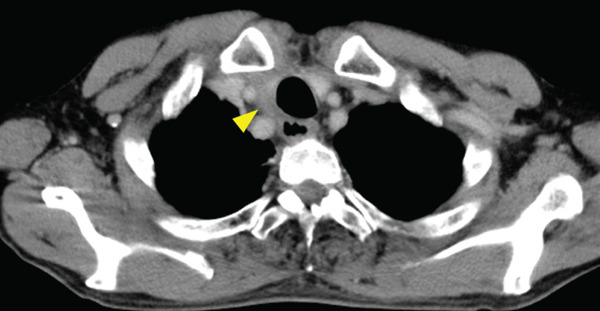
(c)

## 3. Discussion

Although the combination of nivolumab and ipilimumab has shown efficacy in advanced ESCC, early progression within 6 months remains problematic [1, 2]. RT may provide rapid tumor control and simultaneously prime systemic immunity [6], thereby enhancing the effects of ICIs. Preclinical studies have demonstrated that RT increases tumor immunogenicity by inducing immunogenic cell death, enhancing antigen presentation, and promoting dendritic cell activation [9]. RT also upregulates adhesion molecules and chemokines that facilitate T cell infiltration, and activates the cyclic GMP‐AMP synthase–stimulator of interferon genes pathway leading to Type I interferon production [10, 11]. Importantly, when RT is followed by or administered concurrently with ICIs, suppression of tumor growth is observed both within and outside the irradiated field, an effect attributed to synergistic immune activation [7].

The sequence of administration is critical. Tumor‐specific T cells activated by RT infiltrate distant sites within 5–10 days [12, 13]. Thus, RT followed by ICI therapy can effectively amplify RT‐induced systemic immune responses. If anti–PD‐1 therapy is given first, T cells recover from exhaustion and enter proliferation, a state in which they are highly radiosensitive. Subsequent RT may deplete these proliferating T cells, abrogating the expected immune cascade [5]. Experimental data confirm that concurrent or prior RT relative to ICI yields superior antitumor immune responses [14].

Nivolumab plus ipilimumab, which does not cause chemotherapy‐related lymphopenia, may represent an ideal regimen to combine with RT [15]. The dual blockade of PD‐1 and CTLA‐4 activates nonredundant immune pathways, making it theoretically the most promising partner for RT [16]. All lesions in our case were within the radiation field, and thus the abscopal effect was not directly demonstrated. Even so, considering that the median duration of local control and the median progression‐free survival (PFS) for Stage III/IV advanced esophageal cancer treated with RT alone are 7.3/12.1 months and 6.7/5.8 months, respectively [17], the findings from this case study may indicate the additional combination immunotherapy contributed to the favorable outcome.

Clinical evidence also supports this concept. Two randomized Phase II trials in non–small cell lung cancer [18, 19] demonstrated that RT administered before or concurrently with anti–PD‐1 antibody prolonged PFS and showed a trend toward improved OS compared with ICI alone, with pooled analyses reporting a hazard ratio of 0.67 for both PFS and OS [20]. Conversely, trials in head and neck squamous cell carcinoma and other solid tumors did not show any benefit, which is likely due to suboptimal sequencing or low RT completion rates [21, 22]. In Merkel cell carcinoma, ICI monotherapy itself was highly effective, limiting the ability to assess additional RT benefit [23].

The ongoing Phase II trial by the Japan Clinical Oncology Group (JCOG2311) is prospectively evaluating this approach in unresectable or recurrent ESCC, comparing ICI therapy alone with localized RT followed by ICI. In this clinical trial protocol, local irradiation is delivered as 24 Gy in three fractions for solid‐organ lesions of < 3 cm, and 25 Gy in five fractions for lesions in hollow organs or adjacent to hollow organs, as well as for solid organ lesions of ≥ 3 cm. Combination immunotherapy is initiated within 7 days after completion of RT.

In our case, although the treatment response was excellent, pretreatment imaging suggested possible bronchial invasion from cervical lymph node metastasis, indicating noncurative resectability. Thus, proactive salvage surgery was not considered appropriate. The SANO trial investigated an active surveillance strategy after neoadjuvant chemoradiotherapy in patients eligible for curative resection, demonstrating noninferiority to planned salvage surgery [24]. Although our case did not meet surgical eligibility at baseline, the favorable outcome supports the concept that organ preservation and close monitoring may be justified in selected patients, as suggested by SANO. This case may contribute to future treatment strategies for medically inoperable esophageal cancer with favorable response to irradiation and immunotherapy.

## Consent

One patient allowed personal data processing and informed consent was obtained from an individual participant included in the study.

## Conflicts of Interest

The authors declare no conflicts of interest.

## Funding

No funding was received for this manuscript.

## Data Availability

The data that support the findings of this study are available on request from the corresponding author. The data are not publicly available due to privacy or ethical restrictions.
